# Allogeneic Hematopoietic Cell Transplantation for Patients With Deficiency of Adenosine Deaminase 2 (DADA2): Approaches, Obstacles and Special Considerations

**DOI:** 10.3389/fimmu.2022.932385

**Published:** 2022-07-07

**Authors:** Hasan Hashem, Dimana Dimitrova, Isabelle Meyts

**Affiliations:** ^1^ Department of Pediatrics, Division of Pediatric Hematology and Oncology, Bone Marrow Transplant Unit, King Hussein Cancer Center (KHCC), Amman, Jordan; ^2^ Experimental Transplantation and Immunotherapy Branch, National Cancer Institute of the National Institutes of Health, Bethesda, MD, United States; ^3^ Department of Pediatrics, Microbiology, Immunology, and Transplantation, The European Reference Network Rare Immunodeficiency Autoinflammatory and Autoimmune Diseases Network (ERN RITA) Core Center, University Hospitals Leuven, Katholieke Universiteit (KU) Leuven, Leuven, Belgium

**Keywords:** hematopoietic cell transplantation, HCT, deficiency of adenosine deaminase 2, DADA2, inborn error of immunity, bone marrow failure, immunodeficiency, immune dysregulation

## Abstract

Deficiency of adenosine deaminase 2 (DADA2) is an inherited autosomal recessive disease characterized by autoinflammation (recurrent fever), vasculopathy (livedo racemosa, polyarteritis nodosa, lacunar ischemic strokes, and intracranial hemorrhages, end organ vasculitis), immunodeficiency, lymphoproliferation, immune cytopenias, and bone marrow failure. Allogeneic hematopoietic cell transplantation (HCT) is curative for DADA2 as it reverses the hematological, immune and vascular phenotype of DADA2. The primary goal of HCT in DADA2, like in other non-malignant diseases, is engraftment with the establishment of normal hematopoiesis and normal immune function. Strategies in selecting a preparative regimen should take into consideration the specific vulnerabilities to endothelial dysfunction and liver toxicity in DADA2 patients. Overcoming an increased risk of graft rejection while minimizing organ toxicity, graft-versus-host disease, and infections can be particularly challenging in DADA2 patients. This review will discuss approaches to HCT in DADA2 patients including disease-specific considerations, barriers to successful engraftment, post-HCT complications, and clinical outcomes of published patients with DADA2 who have undergone HCT to date.

## Introduction

The deficiency of adenosine deaminase 2 (DADA2), initially described in 2014, caused by biallelic deleterious mutations in the cat eye chromosome region 1 gene (*CECR1*, subsequently renamed *ADA2)*, is a rare autosomal recessive inborn error of immunity disease (1, 2). DADA2 diagnosis is based on an absence or low levels of plasma ADA2 enzymatic activity, and the demonstration of biallelic loss-of-function mutations of *ADA2* (3, 4). DADA2 was initially recognized as a syndrome that manifests with fevers, mild immunodeficiency, polyarteritis nodosa, and early-onset stroke (1, 2, 5, 6). The clinical phenotype has expanded significantly since it was first described in 2014 to also include bone marrow failure (e.g. pure red cell aplasia), autoimmune cytopenias, and liver disease (7–9).

Treatment of DADA2 is challenging and case mortality is estimated to be around 8%, mostly in childhood and related to vasculopathy-associated complications and infections (10–13). Immunosuppressants such as corticosteroids are often the choice for initial treatment efforts, albeit with variable success. Other agents such as azathioprine, cyclosporine, tacrolimus, mycophenolate mofetil, cyclophosphamide, and methotrexate have all been used depending on phenotype specifics, but with little or transient effect. At present, the mainstay of treatment of vasculopathy and autoinflammation phenotypes consists of anti-TNF-agents (etanercept, infliximab, adalimumab) with impressive reductions in stroke incidence (14, 15). Nevertheless, anti-TNF-agents were not effective or have shown only modest control of the hematological and severe immunodeficiency phenotypes (14, 16). Moreover, breakthrough inflammation has also been described (14). In these patients, HCT is the only curative treatment until present (17, 18).

## Pathophysiology of DADA2

The pathophysiology of DADA2 remains unclear but increased neutrophil extracellular trap formation (NETosis) and reduced M2 macrophage differentiation have been proposed (1, 19, 20). ADA2 is predominantly expressed in monocytes, and the immunological profile of DADA2 patients indeed shows a polarization toward proinflammatory M1 macrophages (21–23). Activation of multiple inflammatory responses, including type I and II interferon (IFN) pathways and production of the proinflammatory cytokines IL-1B, IL-6, and TNF-alpha, in turn results in tissue damage and reduced endothelial integrity (20, 21, 24–28).

## Understanding DADA2 Phenotypes

At present time, more than 240 patients have been reported in the literature (10, 11, 29–31). The onset of disease is usually in childhood but adult-onset has also been described with the oldest patients reported at 59 years of age (27, 31, 32). Presentations are highly heterogenous, rendering early diagnosis difficult. Even within single kindreds, there is tremendous variability in DADA2 phenotypes and sometimes incomplete or variable penetrance (4). Some insight into a potential genotype-phenotype correlation was provided by Lee et al. who suggested that the phenotype is dependent on the residual ADA2 enzyme activity function, as measured in the supernatant of a HEK293T overexpression model (16).

Since DADA2 was first described in 2014, the phenotype has been extended significantly to include not only vasculopathy and autoinflammation but also immunodeficiency and hematologic manifestations. Vasculopathy of small- and medium-sized arteries is a major clinical feature of DADA2 with skin and central nervous system most commonly involved, ranging from livedo racemosa and polyarteritis nodosa (PAN) to intracranial vasculopathy with lacunar ischemic strokes and hemorrhages. Autoinflammation with recurrent fevers and elevated CRP and ESR are also reported in more than half of patients (31). Recurrent bacterial and viral infections have been described, with exceptional susceptibility to herpes virus infection, especially CMV and EBV (33–36). Laboratory evaluations may show lymphopenia, hypogammaglobulinemia, and decreased memory B-cells (32, 37). Hematological manifestations, particularly as an indication for HCT, have been increasingly appreciated in recent years. Pure red cell aplasia (PRCA) was described initially in three patients and further confirmed by additional reports (7, 8, 38). Other patients present with neutropenia, thrombocytopenia, pancytopenia, and autoimmune cytopenias (8, 17, 18, 39). Rarely, some DADA2 patients can present with hemophagocytic lymphohistiocytosis (HLH). Bone marrow biopsies show reticulin fibrosis and characteristic lymphoid aggregates (13, 31). Lymphoproliferation is another important feature; autoimmune lymphoproliferative syndrome-like presentation, clonal lymphoproliferation, T-large granular lymphocytosis, and lymphoma have all been described (40–42).

## HCT Indications, Treatment and Outcomes

Currently, there are 36 patients who underwent 45 HCT for DADA2 in 15 countries and 24 centers (18, 31, 43, 44). Indications for HCT included PRCA, neutropenia, combined RCA and immune-mediated neutropenia, pancytopenia, autoimmune hemolytic anemia (AIHA), diffuse large B-cell lymphoma (DLBL), immune dysregulation, severe aplastic anemia, and severe lymphopenia (**Table 1**).

**Table 1:** HCT characteristics and outcomes for published DADA2 patients .

HCT Variable		Number of patients (n=36)	Percentage of patients (%)	Graft failure (n=4)	Mortality (n=2)
Year of HCT	<2015	8	22		
	>2015	28	78	4	2
Age at HCT	<18	28	78	3	2
	>18	8	22	1	
Gender	M	14	39	2	
	F	22	61	2	2
Donor	MRD	5	15		1
	10/10 MUD	18	55	2	
	9/10 MUD	8	22	1	1
	Haplo	2	5		
	7/8 cord	1	3	1	
	Unknown	2			
Graft source	BM	25	72	3	2
	PB	9	28	1	
	Unknown	2			
Conditioning intensity	MAC	24	70	1	2
	RIC	10	30	3*	
	Unknown	2			
Indication for HCT	PRCA	10	28		1
	Neutropenia	10	28	2	
	RCA/neutropenia	6	18	2	
	Pancytopenia	3	8		
	Others	7	20		1

BM: bone marrow; F: female; HCT: hematopoietic cell transplantation; M: male; MAC: myeloablative conditioning; MRD: HLA-matched related donor; MUD: HLA-matched unrelated donor; PB: peripheral blood; PRCA: pure red cell aplasia; RCA: red cell aplasia; RIC: reduced intensity conditioning. *: of the 3 GF in RIC recipients, one received serotherapy-free regimen. The other 2 received serotherapy but one of them received cord blood

All but 2 of the patients are alive and well and are cured at a median follow-up of two years with an overall survival of >95%. The majority of patients were transplanted after 2015 and were children. Almost half of HCTs were from HLA-matched unrelated donors. Myeloablative conditioning and bone marrow as a graft source constituted more than two thirds of HCTs (**Table 1**).

## Approaches to HCT for DADA2

### Bridge to HCT Considerations

Anti-TNF agents are the mainstay of treatment of vasculopathy and autoinflammation phenotypes and it is recommended that patients already on these agents, who show some response, continue therapy until conditioning for HCT starts, even until engraftment. This might help decrease inflammation associated with DADA2 and decrease peri-transplant complications. Moreover, some patients withDADA2 especially those with liver disease and/or iron overload due to frequent blood transfusions, might benefit from iron chelation prior to HCT.

### Preparative Regimens

The choice of preparative regimen should first be guided by the patient’s disease phenotype and end-organ status (**Table 2**). Previous cohort reports have pointed to a specific hepatic vulnerability in DADA2 patients, with some patients developing chronic liver disease post-HCT even in the absence of clinically overt liver disease prior to HCT especially in patients with PRCA who tend to have iron overload due to previous frequent blood transfusions (17, 18, 31). A careful work-up of the patient, including at least transient elastography, imaging, or preferably, liver biopsy, seems mandatory to adequately assess the status of the liver, going into HCT. Depending on the availability of specific drugs, a less toxic regiment may be used, such as treosulfan instead of busulfan. Also the combination of sirolimus as a GvHD prophylaxis after busulfan conditioning should be avoided (45, 46). Center-specific sinusoidal obstruction syndrome prophylaxis should be adhered to (ursodiol, relative fluid restriction and defibrotide if available). For DADA2 patients who present with HLH, specific conditioning regimens similar to the ones used for HLH might be advised. It is also important to perform a careful assessment of other end organs, including renal imaging and function assessment (microalbuminuria, proteinuria) esp. given the added renal toxicity of most immunosuppressants and of anti-viral agents.

**Table 2:** DADA2 specific-disease vulnerabilities.

Disease-specific vulnerability	Potential impact of preparative regimen	Agents to be used with caution
Vasculopathy of -small and medium-sized arteries and/or history of strokes/ICH	Peri-transplant and peri-engraftment bleeding	Myeloablation, particularly busulfan-based (PK monitoring can aid in reducing toxicity), TBI
Autoinflammation with fever and elevated inflammatory markers	Peri-transplant inflammation and cytokine storm	Myeloablation, particularly busulfan-based (PK monitoring can aid in reducing toxicity), TBI high-dose cyclophosphamide
Iron overload and red cell alloimmunization	SOS, higher transfusion needs	Myeloablation, particularly busulfan-based (PK monitoring can aid in reducing toxicity), TBI high-dose cyclophosphamide
Liver disease (hepatitis, hepatomegaly, hepatoportal sclerosis, portal HTN, iron accumulation)	SOS	Myeloablation, particularly busulfan-based (PK monitoring can aid in reducing toxicity), TBI high-dose cyclophosphamide
Preexisting infection/severe immunodeficiency and prolonged antibiotics usage	Disruption of mucosal barrier and microbiota dysbiosis	Myeloablation, particularly busulfan-based (PK monitoring can aid in reducing toxicity), TBI high-dose cyclophosphamide
Prolonged severe neutropenia	Susceptibility to fungal infections	Myeloablation, particularly busulfan-based (PK monitoring can aid in reducing toxicity), TBI high-dose cyclophosphamide
Lymphoid aggregates in the bone marrow and autoimmune cytopenias	Graft failure	Reduced intensity conditioning, particularly in absence of serotherapy

PK, pharmacokinetics; SOS, sinusoidal obstruction syndrome; TBI, total body irradiation.

From the reports thus far, myeloablative conditioning regimens have been most commonly used although reduced intensity conditioning has also been successfully used. The most commonly used regimen was treosulfan/fludarabine +/- thiotepa with rabbit ATG or alemtuzumab, reflecting protocols A, B, C, D from a recent IEWP-EBMT update (47). Most patients transplanted received serotherapy, with the choice for either rabbit ATG or alemtuzumab mostly dependent on the center preference. In the context of serotherapy and given the specific vulnerability to viral infections esp. herpesvirus infections, in DADA2, careful monitoring of viremia (EBV, CMV, Adv, HHV6) and prophylaxis when available are advised.

Potentially more important than any other consideration is that these HCTs should be performed by an experienced HCT team, in a center with the necessary HCT expertise available, as well as the necessary logistics such as quick turnaround chimerism analysis etc. An equally important consideration is the timing of HCT –given the risk of life-threatening mortality and morbidity associated with this condition, we suggest that, as with other inherited disorders of immunity, HCT early in life for patients with severe phenotypes is preferred before organ damage has evolved, notwithstanding successes of adolescent and adult HCTs.

### Donor Selection

All related donors under consideration must be screened for *ADA2* mutations, even if they appear clinically healthy. Prior to elucidation of disease genetics, at least two known HCT procedures were performed using donors subsequently found to have biallelic pathogenic mutations (17, 18). Ideally, plasma ADA2 levels should also be checked (3, 48).

Because DADA2 is inherited in an autosomal recessive fashion, mutation-negative related donor options are often scarce. Thus, the question of whether to prioritize a matched unrelated donor over a carrier matched sibling, or whether to use a heterozygous related donor at all in the absence of unrelated donor options, arises frequently. Evaluations of subjects with monoallelic mutations have shown significant variability. Most are healthy with a plasma ADA2 concentration intermediate between a healthy control and a patient with biallelic *ADA2* defects, but clinically affected carriers with both detectable ADA2 and undetectable ADA2 levels have been described (1, 48–50). Furthermore, adult onset or mild disease manifestations have been described in carriers, along with *in vitro* abnormalities such as enhanced NET formation, suggesting the potential for an immunological phenotype that may not be readily apparent in a young prospective donor (20, 50).

Utilizing a heterozygous donor therefore raises theoretical concerns regarding the possibility of developing disease manifestations over time, even in the context of full donor chimerism. Furthermore, because the donor chimerism sufficient for phenotype reversal is unknown, recipients of a graft from a carrier donor could be more prone to developing disease manifestations in the setting of mixed chimerism. Even using an unaffected donor, mixed whole blood chimerism up to 30% donor was associated with a decline in plasma ADA2 enzyme activity and new hematological DADA2 disease manifestations in one patient (17, 18, 51).

Four HCTs have been performed using a clinically healthy donor with a confirmed monoallelic *ADA2* mutation. In one, bone marrow collection yielded insufficient CD34+ cell counts (0.5x10^6/kg), requiring a subsequent stem cell boost, while bone marrow collection yields were as expected in the other case. These two patients are clinically well with full donor chimerism and without major complications at 3 and 6 years post-HCT. A third patient died, with further details not available (17, 18). A fourth patient received a salvage HCT from her carrier mother after prior graft failure and has continued reversal of disease phenotype in the setting of full donor chimerism >2 years later (31). Given a risk of graft instability, particularly in DADA2 patients with immune-mediated neutropenia, donor selection must also take into consideration factors related to donor availability and logistics, as unplanned donor cell infusions may be necessary to prevent graft rejection (17, 18, 31).

### Anticipating and Mitigating Graft Instability

Of 36 known patients who received HCT for DADA2 to date, graft failure occurred in four patients (primary, n=1; secondary, n=3), while declining chimerism was observed in two patients despite myeloablative conditioning (18, 31). All three patients with secondary graft failure had neutropenia as an indication for HCT, associated with increased T cell infiltration in the bone marrow along with a myeloid maturation arrest at baseline. Remarkably, two of these patients had continued graft instability even after subsequent HCT, manifested by repeated rapid declines in donor T cell chimerism (0% and 9% with myeloid chimerism of 60% and 78%, respectively), coinciding with development of dense neutropenia and necessitating prompt immunosuppression as well as multiple donor lymphocyte infusions. In all three cases, graft stabilization was tied to development of graft-versus-host disease with presumed concurrent graft-versus-marrow effect (31). Interestingly, a fourth patient, who experienced declining donor chimerism (30% whole blood, 0% CD3) rescued with two stem cell boosts, developed new agranulocytosis as ADA2 levels declined in the context of a viral reactivation. Further details to confirm whether host T cells were implicated as in the other patients are not available. A fifth patient, who was transplanted for pure red cell aplasia without neutropenia, tolerated a decline in donor chimerism to 55% without disease manifestations or need for subsequent cell infusion (18).

Thus, a subset of DADA2 patients with T-cell-mediated neutropenia may be more prone to graft rejection and should be counseled accordingly, taking this risk into consideration in planning the most appropriate HCT platform, graft, and post-HCT monitoring. In clinical practice, these HCT candidates may have a history of neutropenia responsive to T lymphocyte-targeted immunosuppressive methods, such as high-dose corticosteroids or cyclosporine. Serotherapy-containing conditioning platforms resulting in robust lymphodepletion are most effective for such patients, while myeloablation is not protective and likely not necessary, and may be particularly harmful in a patient population that is already vulnerable to endothelial damage and hepatic complications. Other strategies aimed at reducing graft rejection may include utilizing T-cell replete grafts, peripheral blood stem cell grafts, and/or higher graft doses. Given the potential for extremely rapid graft rejection, donor chimerism including T cell chimerism should be monitored closely and frequently post-HCT. Unexpected declines in neutrophil count should raise concern, and augmenting immunosuppression may be considered to stall resurgence of host T cell-mediated destruction. Ideally, HCT planning should include a contingency plan to ensure prompt collection and infusion of additional donor cells can be readily arranged if threatened graft failure is identified, although certain patients may benefit from additional donor cell infusion (lymphocytes or stem cell boost, depending on the clinical scenario) or even require a second HCT using a different donor.

## Conclusions

HCT is a potentially life-saving and definitive treatment for DADA2 as it reverses the hematological, immunological and vascular phenotype of the disease. Most HCT procedures reported thus far were performed in children with a bone marrow failure phenotype. Overall survival is excellent, but graft failure is a frequent complication, and optimizing immunoablation through use of serotherapy containing regimens may be beneficial. Careful attention to the presence of end-organ disease, particularly liver dysfunction, is warranted in all DADA2 patients but especially for those undergoing HCT. Continued follow-up of DADA2 patients receiving HCT will aid us in defining the most appropriate HCT regimens in DADA2.

## Author Contributions

HH, DD, and IM designed the study, reviewed the literature, and wrote the manuscript. All authors contributed to the article and approved the submitted version.

## Funding

IM is a Senior Clinical Investigator at the FWO – Flanders, and is supported by the KU Leuven C1 grant C16/18/007, by a VIB GC PID Grant, by the FWO Grants G0B5120N (DADA2) and G0E8420N and by the Jeffrey Modell Foundation. This project has received funding from the European Research Council (ERC) under the European Union’s Horizon 2020 research and innovation programme (grant agreement No. 948959).

## Conflict of Interest

The authors declare that the research was conducted in the absence of any commercial or financial relationships that could be construed as a potential conflict of interest.

## Publisher’s Note

All claims expressed in this article are solely those of the authors and do not necessarily represent those of their affiliated organizations, or those of the publisher, the editors and the reviewers. Any product that may be evaluated in this article, or claim that may be made by its manufacturer, is not guaranteed or endorsed by the publisher.
